# Syphilis of the Nervous System

**Published:** 1871-01

**Authors:** 


SYPHILIS OF THE NERVOUS SYSTEM.—Dr. E. L. Keyes, of New York City, contributes to the *N. Y. Medical Journal* a paper forty pages long, on nervous syphilis, based on 34 cases treated by himself and Dr. VanBuren. The cases were all in private practice. Many of them presented several nervous symptoms at the same time, but, naming them by the most prominent symptoms, there were 14 cases of hemiplegia, 9 of paraplegia, 4 of epilepsy, 2 of facial paralysis, 1 of paralysis of biceps and deltoid, and 4 of intellectual derangement. There have been 11 recoveries, 10 have improved, 7 have died, and 6 have been lost trace of.

After detailing the histories of these several cases, and after extended comments upon them, the writer sums up as his conclusions from the study substantially as follews: Nervous symptoms depending upon syphilis may occur at any time during the life of the individual after a few weeks subsequent to the infecting chancre. The earlier nervous symptoms (paralytic or otherwise) occur, the less likely is there to be any lesion which an autopsy can reveal; and there is no constancy between the character of the lesion and that of the nervous symptoms to which it gives rise.

The pathology of the nervous symptoms in many cases is, doubtless, cerebral congestion. Syphilitic hemiplegia usually occurs without loss of consciousness, the paralysis usually coming on gradually, the patient being under 40, and having had fixed, constant headache for some time before the attack.

Mydriasis, existing alone, or with other nervous symptoms, and without positive disease of the eye, is presumptive evidence of syphilis.

Paralysis of single muscles, or sets of muscles, are frequently syphilitic.

Syphilitic paralysis usually comes on gradually, and is rarely complete; the bladder almost always suffers more or less from it, and requires local treatment.

Syphilitic epilepsy generally occurs after the thirtieth year, there having been nothing of the sort in early life; the attack is usually proceeded by headache. The convulsions occur often, the intermission between the series of attacks being long, but during this period the nervous symptoms usually become exaggerated.

Loss of memory is a common symptom, as also are mental disturbances, from mild illusions to insanity. Aphasia is often associated with the intellectual disturbances. Inordinate emotional expressions are often associated with the mental weakness of syphilis.

The prognosis is better when the nervous symptoms are caused by syphilis than when like symptoms are due to an equally extensive lesion from another cause, but after arrest of the disease, there is often left an impression, causing incurable impairment of function. The best treatment is large doses of iodide of potassium, (a drachm or two per day). Mercury at the same time, or alternated with the iodide, is often valuable in protracted cases. Tonics, and change of air and surroundings are always valuable.

